# Partial Discharge Monitoring in Power Transformers Using Low-Cost Piezoelectric Sensors

**DOI:** 10.3390/s16081266

**Published:** 2016-08-10

**Authors:** Bruno Castro, Guilherme Clerice, Caio Ramos, André Andreoli, Fabricio Baptista, Fernando Campos, José Ulson

**Affiliations:** Faculdade de Engenharia, UNESP-Univ. Estadual Paulista, Bauru, Departamento de Engenharia Elétrica, Av. Eng. Luiz Edmundo C. Coube 14-01, 17033-360 Bauru–SP, Brazil; bruno.castro@feb.unesp.br (B.C.); marabezzi@feb.unesp.br (G.C.); cramos@feb.unesp.br (C.R.); andreoli@feb.unesp.br (A.A.); fcampos@feb.unesp.br (F.C.); ulson@feb.unesp.br (J.U.)

**Keywords:** partial discharge, piezoelectric sensors, low-cost, acoustic emission, transformer, dielectric

## Abstract

Power transformers are crucial in an electric power system. Failures in transformers can affect the quality and cause interruptions in the power supply. Partial discharges are a phenomenon that can cause failures in the transformers if not properly monitored. Typically, the monitoring requires high-cost corrective maintenance or even interruptions of the power system. Therefore, the development of online non-invasive monitoring systems to detect partial discharges in power transformers has great relevance since it can reduce significant maintenance costs. Although commercial acoustic emission sensors have been used to monitor partial discharges in power transformers, they still represent a significant cost. In order to overcome this drawback, this paper presents a study of the feasibility of low-cost piezoelectric sensors to identify partial discharges in mineral insulating oil of power transformers. The analysis of the feasibility of the proposed low-cost sensor is performed by its comparison with a commercial acoustic emission sensor commonly used to detect partial discharges. The comparison between the responses in the time and frequency domain of both sensors was carried out and the experimental results indicate that the proposed piezoelectric sensors have great potential in the detection of acoustic waves generated by partial discharges in insulation oil, contributing for the popularization of this noninvasive technique.

## 1. Introduction

Partial discharges (PD) are a random phenomenon that depend on the applied electric field, being that the presence of electrical charges generally causes deterioration of the electric insulation of power transformers [1,2]. Some critical factors in the operation of transformers, such as overload, nonlinear loads, transient voltage surges by atmospheric origin, and switching, can make the insulation system of transformers to lose their physical and chemical properties. Therefore, these operating conditions can cause early deterioration of the insulation, allowing the occurrence of internal partial discharges that can become major defects and, consequently, decrease the useful life of power transformers.

Online monitoring of PD can reduce the risk of failure of high voltage and high power transformers due to damage caused in insulation system [3,4,5,6]. There are many established methods for identification of partial discharges in high voltage devices, such as the electrical method, chemical method, and acoustic wave propagation for PD identification. The choice of the appropriate method depends on the type of equipment being monitored. As is well known, the method based on the acoustic wave propagation, commonly known as the acoustic emission (AE) method, has the advantage of being non-invasive to the monitored equipment, i.e., are non-destructive testing (NDT) methods.

The principle of the AE method is based on detecting acoustic waves generated when PDs occur in the transformers. These acoustic waves are detected by AE sensors fixed on the external wall of the transformers [6,7,8,9]. However, the main disadvantage of this method is the high cost of the commercial AE sensors, especially in applications where multiple sensors are required. In this context, the objective of this paper is to present a low-cost noninvasive method for PD detection in power transformers by applying low-cost piezoelectric diaphragms, commonly known as “buzzers”.

Based on the AE method, a low-cost piezoelectric sensor attached to the external wall of a transformer is able to detect the elastic waves emitted by PDs, allowing non-invasive PD monitoring. As a result, online continuous monitoring for defect prevention contributes decisively to the improvement in the quality of electricity supply. Moreover, considerable material losses can be prevented, improving the equipment reliability, safety, and availability.

A comparative study between the low-cost piezoelectric sensor and a commercial AE sensor, which is consolidated in the scientific and industrial community, is presented in this paper. Under the experimental conditions considered in this study, the results show that the proposed low-cost piezoelectric sensor for PD detection based on the AE method can be a low-cost real alternative compared the high-cost commercial AE sensor.

The remainder of this paper is organized as follows: Section 2 presents a brief theory about PDs and introduces the AE method in power transformers; Section 3 shows the piezoelectric sensors used in this study; Section 4 presents the material and methods; Section 5 discusses the results; and finally, the conclusions are presented in Section 6.

## 2. Partial Discharges

The PDs occur when an electric field incident in the material undergoes significant changes that produce an electric current in specific area partially closing the circuit. According to the IEC 60270 standard [10], PDs are defined as “localized electrical discharges that only partially bridges the insulation between conductors and which can or cannot occur adjacent to a conductor”. Furthermore, the PDs are often a consequence of an electric field and charges on the discontinuity surface or voids present within a dielectric material. Voids can cause a local electric field enhancement and, subsequently, originate PDs. Usually, the PDs appear as pulses with a duration of less than 1 ms, emitting light, heat, acoustic and electromagnetic waves [4,5,6,7]. 

There are two consolidated models that describe the behavior of PDs in alternating voltages. In the first model, after the extinction of the discharge in a void, the voltage on this same point starts to increase again until there is another PD when the threshold is reached, given by the superposition of the applied electric field and the field formed by residual surface charges left by the last PD [11,12,13,14,15]. The second model considers one uniform electric field produced by alternating voltage in a spherical void surrounding a dielectric material. The electric field varies proportionally to the applied alternating voltage. The charges released by the first PD are directed at the walls of the void, in which the local field is the sum of the field applied on the electrode, and when the total field reaches the threshold, a PD can appear. Thus, the PD is exclusively a function of an electric field intensity in the void, according to this approach [11].

The PDs are related to the condition of insulating materials in transformers. According to Boczar [16], natural or premature degradation of the physicochemical properties of the insulating oil can result in the formation of gas bubbles in the medium insulating material. Moreover, the author defines eight kinds of PD groups, including the point-to-point discharges in the insulating oil, which are the PD analyzed in this study.

The PD monitoring by AE is based on the detection of elastic waves emitted from the PD source. The insulation systems failure in power transformers can cause internal PDs. For the insulation materials, one discontinuity (or void) can cause the local electric field enhancement and, consequently, originate PDs. The PD is characterized by small electric sparks that partially close the circuit between the conductive parts of the insulating material. The phenomenon causes a local disturbance, emitting heat, light, electromagnetic radiation, and acoustic pressure waves in the form of burst/impulsive pulses, which are radiated in all directions from the discharge source. Since part of the energy is released in the form of acoustic waves, the PD monitoring using the AE method is based on detecting these elastic waves [10,11,12,13,14,15,16,17,18]. 

The elastic waves, with a frequency range from 20 kHz to 1 MHz, are classified according to the propagation modes. There are four types: longitudinal, shear, surface, and plate waves. For the shear waves, the movement is transverse to the direction of propagation. A surface wave describes an oscillating movement with an elliptical orbit and a plate wave describes an oscillating particle movement in very thin plates. The liquid acoustic propagation medium is mainly characterized by the presence of longitudinal waves. In this case, the movement occurs entirely in the direction of the wave propagation. Therefore, the PD in the mineral oil of power transformers emits longitudinal waves in this insulating material. 

Metal plates, such as a transformer tank, support both types: longitudinal and shear waves [17]. When a PD occurs, the longitudinal waves travel through the mineral oil. The waves suffer refraction through the surface of the steel tank, yielding longitudinal and shear waves [17]. Accordingly, these refracted waves in the steel surface of the tank can be detected using AE sensors. The major advantage of this method is to allow the monitoring of PDs with the transformer in constant service [4,12]. The approach in this paper is to support the AE technique with low-cost piezoelectric sensors, such as piezoelectric diaphragms, commonly known as “buzzers”.

## 3. Piezoelectric Sensors

The piezoelectric materials are increasingly popular and can be used as sensors and actuators [19]. The piezoelectric effect occurs in materials that, when subjected to a tensile stress, produces a voltage output through the formation of an electric dipole in the material. The reverse effect also occurs, applying an electrical voltage between the two sides of the piezoelectric material a mechanical deformation arises.

Therefore, there is an interaction between the electrical and mechanical quantities of the piezoelectric material. Disregarding the thermal and magnetic effects, and considering only the linear piezoelectricity, the basic constitutive equations [19] for the direct and reverse piezoelectric effects are given by Equations (1) and (2), respectively:

(1) $$D_{i}=d_{ikl}T_{kl}+\varepsilon_{ik}^{T}E_{k}$$

(2) $$S_{ij}=s_{ijkl}^{E}T_{kl}+d_{kij}E_{k}$$

where $d_{ikl}$ and $d_{kij}$ are the piezoelectric constants, $\varepsilon_{ik}^{T}$ is the permittivity component at constant stress, $D_{i}$ is the electric displacement component, $E_{k}$ is the electric field component, $s_{ijkl}^{E}$ is the elastic compliance constant at constant electric field, $S_{ij}$ is the strain component, $T_{kl}$ is the traction vector component, and *i*, *j*, *k*, *l* = 1, 2, 3.

According to the constitutive equations, in a piezoelectric material there is an electric load due to a mechanical stress and a deformation due to an electric field, i.e., there is an electromechanical coupling. Therefore, the piezoelectric sensors have the property of generating a voltage when excited by a mechanical deformation that is generated by an acoustic wave. This property is used in this study to monitor AE signals generated by PDs.

**Figure 1:**
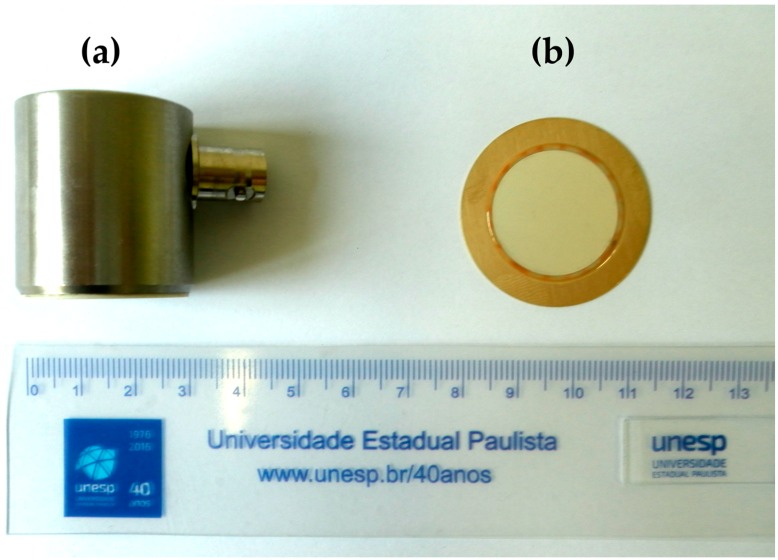
Sensors used in this study: (**a**) conventional RS15I-AST sensor [20] consolidated in AE applications; and (**b**) alternative low-cost piezoelectric diaphragm (buzzer) [21].

Normally, the industrialized sensors have a high average cost, ranging from hundreds to thousands of dollars, such as the RS15I-AST sensor [20] from Physical Acoustics-MISTRAS Group (Princeton Jct., New Jersey, NJ, USA), which is consolidated for AE applications and is used in this study as a reference, as shown in Figure 1a. This sensor is enclosed in a metal housing to minimize interferences and incorporates a low-noise input preamplifier and a filter all inside the sensor housing.

As a proposal of an alternative low-cost sensor, this study presents an experimental analysis of the applicability of piezoelectric diaphragms in the monitoring of PDs based on the AE method. Figure 1b shows a piezoelectric diaphragm, which is commonly known as “buzzer”.

Piezoelectric diaphragms [21] are sound components with a simple structure consisting of a piezoelectric ceramic disk adhered to a brass plate, as shown in Figure 1b. The ceramic is coated with a metal film that serves as an electrode. These components are manufactured by several vendors, such as Murata Manufacturing (Nagaokakyo-shi, Kyoto, Japan), and are readily available at a low cost, ranging from cents a few dollars depending on the size and manufacturer.

The basic application of piezoelectric diaphragms has been the generation of sound such as alarm and beep in various electronic devices. However, due to their low cost, these sound components have been used in many scientific studies on various applications and good results have been reported [22,23,24]. In the current study, the objective is to analyze the effectiveness of piezoelectric diaphragms [21] for detecting PD in power transformers. Experiments were performed in a power transformer and the results were compared with the conventional AE sensor [20] using time and frequency parameters. As is well known, piezoelectric sensors are significantly sensitive to temperature variations [25,26]. However, the temperature and other interferences are not considered in this study. The materials and methods are presented in the next section.

## 4. Materials and Methods

The low-cost piezoelectric sensor used in this study is an equivalent model to the 7BB-35-3 piezoelectric diaphragm from Murata Manufacturing [21] with ceramic disk of 25.0 mm × 0.23 mm and brass plate of 35.0 mm × 0.30 mm, as previously shown in Figure 1b. As a reference sensor for the comparison of the results we used the conventional AE sensor RS15I-AST from Physical Acoustics-MISTRAS Group [20], as shown in Figure 1a.

### 4.1. Initial Characterization

Since the object of this study is to analyze the feasibility of application of an alternative low-cost sensor for identification of PDs in power transformers based on the AE method, an initial analysis between the voltage signals generated by the piezoelectric diaphragm and the conventional AE sensor was carried out to compare the performance of them. The frequency response of these two sensors was characterized using the pencil lead break (PLB) method.

The PLB method is regulated by the E976-10 standard [27] and commonly used to characterize AE sensors [28]. Recently, this method has also been used to characterize other types of piezoelectric sensors [29]. The procedure consists in breaking a pencil lead against the structure or rod on which the sensor is installed. Breaking the pencil lead, an impulsive stress is released and, consequently, a wide-band signal is generated which can be used to evaluate the sensor response.

The conventional AE sensor and the piezoelectric diaphragm were fastened on a steel plate of 200 mm × 200 mm × 3 mm using liquid paraffin, allowing to perform the PLB test using a mechanical pencil provided with a lead with length of 3 mm and diameter of 0.5 mm. While the lead was broken, the signal voltage from the sensors have been sampled with a sampling rate of 1 MS/s using an oscilloscope. The analysis was performed in the frequency domain computing the power spectral density (PSD).

### 4.2. Partial Discharge Monitoring

**Figure 2:**
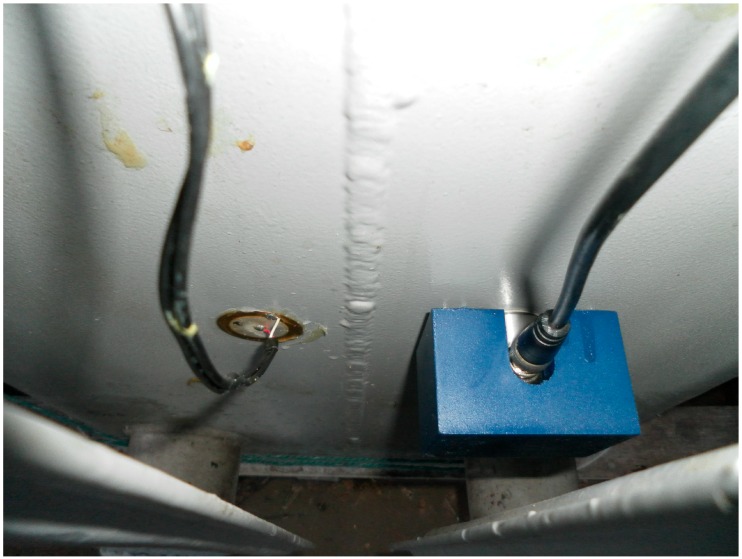
Experimental setup.

The piezoelectric diaphragm and the conventional AE sensor were fastened in the transformer’s tank of approximately 270 × 540 × 720 mm using liquid paraffin to analyze the PD signals. In addition, a magnetic holder was used in the conventional AE sensor mounting. The experimental setup is shown in Figure 2.

**Figure 3:**
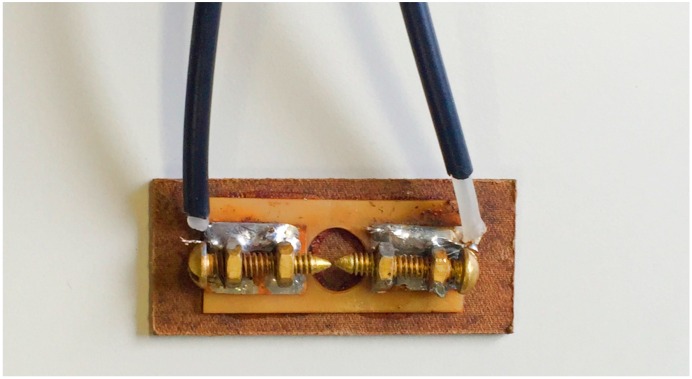
Electrodes used to generate PDs.

Partial discharges were generated in the transformer oil by applying a voltage of 7 kV in a copper electrode, as shown in Figure 3.

An amplifier with frequency response up to 500 kHz and gain of approximately 28 dB was used simultaneously as an antialiasing filter and to amplify the signal from the piezoelectric diaphragm. In addition, a high-pass filter with cutoff frequency of 20 kHz was used to eliminate low frequencies deriving from environmental vibrations. The data acquisition was performed using an oscilloscope with sampling rate set to 1 MS/s. The conventional AE sensor incorporates in its housing an amplifier and filter, and did not require additional signal conditioning.

The time and frequency parameters of both sensors obtained using the proposed methodology were considered in this paper. The signal processing parameters are presented in the next section.

### 4.3. Signal Processing Parameters

**Figure 4:**
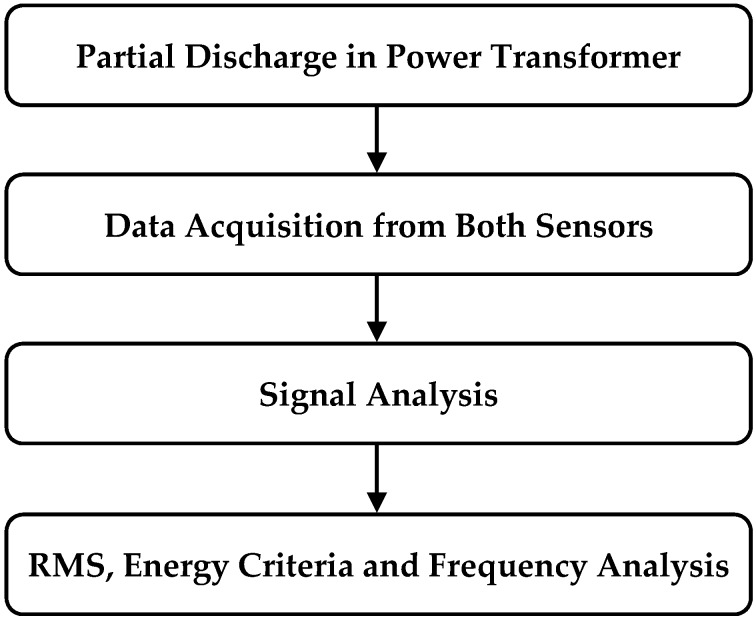
Signal analysis flowchart.

The PD’s acoustic emission data provided by the oscilloscope allow performing analysis in the frequency domain using several routines developed in MATLAB from MathWorks, Inc. (Natick, MA, USA), such as the fast Fourier transform (FFT) algorithm and power spectral density (PSD). The statistical parameters used for the time analysis are related to the load intensity applied in the sensors or even temporal parameters. This study uses the energy criterion, root mean square (RMS), and time of arrival (TOA) as statistical parameters. These parameters are powerful tools for response analysis of the sensors for PD phenomenon. Figure 4 shows the flowchart performed for the signal analysis.

#### 4.3.1. Energy Criterion

The energy criterion describes AE signals predominantly characterized by the variation of the frequency spectrum and energy content. Furthermore, this criterion is a technique to detect the arrival time and changes in the AE signal [30]. 

The partial energy curve is defined as the cumulative sum of amplitude values of the AE signal, according to Equation (3):

(3) $$S(i)=\sum_{k=0}^{i}\left(x_{k}^{2}-\frac{i\cdot S_{N}}{N}\right)$$

where *S(i)* is the partial energy of the sampled signal x, *i* is the number of samples of a selected part of the signal, *S_N_* represents the total energy and *N* is the length of the signal. Hence, the negative trend is dependent on a total energy *S_N_* and the number of samples *N*. The energy curve features a global minimum, which corresponds the arrival time of the AE signals. This criterion is important to characterize how the acoustic waves affect the piezoelectric diaphragm and AE sensor [30].

#### 4.3.2. Root Mean Square (RMS) Criterion

There are many types of signal evaluation can be applied to the output of the sensors, being the RMS criterion is one of the most important for this task. This approach is directly related to the load applied to the sensor and is an attractive attribute for any monitoring application. Based on the formal definition of the RMS value, the RMS parameter for analysis of finite time AE signals [31] is given by Equation (4):

(4) $$x_{RMS}=\sqrt{\frac{1}{T}\int_{t}^{t+T}{\left[x(t)\right]}^{2}dt}=\sqrt{\frac{1}{N}\sum_{i=1}^{N}x_{i}^{2}}$$

where *T* is the integration time (*T* was considered equal to 1 ms in this paper) and *N* is the discrete number of AE data (samples) within the *T* interval. Thus, the criterion can show the behavior of both sensors used in this paper, allowing perform a correlation signal analysis.

## 5. Results and Discussion

This section presents the results obtained from tests for PD detection using low-cost sensors for the methodology proposed in this study.

### 5.1. Initial Characterization

The initial characterization using the PLB method performed in the steel plate provides the behavior of the piezoelectric diaphragm and the conventional AE sensor in the frequency domain, being that this test verifies the feasibility of the diaphragm to detect PDs. Although the PLB test is very different from a PD, this test serves to perform a preliminary study of a particular application, considering that the responses of the signals are similar.

**Figure 5:**
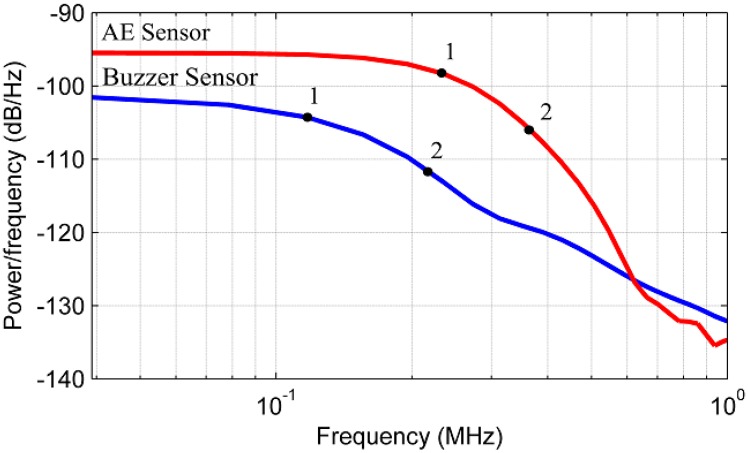
PSD obtained for the piezoelectric diaphragm (buzzer) and the conventional AE sensor.

Tests were carried out and the experimental results were analyzed. Figure 5 shows the PSD obtained for the sensors and Table 1 shows the highlighted points in the PSD analysis. These points were chosen only to compare the two PSDs. According to Figure 5, the conventional AE sensor has a higher PSD in almost the entire frequency range tested, indicating a higher sensitivity, except for frequencies above approximately 600 kHz, wherein the piezoelectric diaphragm (buzzer) has shown a higher sensitivity. In addition, the points highlighted in Figure 5 and shown in Table 1 indicate that the diaphragm sensor has 3 dB attenuation at 120 kHz and 10 dB attenuation at the 215 kHz. In contrast, the conventional AE sensor has 3dB attenuation at 237 kHz and 10 dB attenuation at 357 kHz.

Although the diaphragm sensor does not have the same frequency response compared to the conventional AE sensor, this sensor presents a wide spectrum range for the PLB test, indicating higher sensitivity than the conventional sensor for frequencies above approximately 600 kHz, although the PSD is very low (less than −125 dB) for both sensors at high frequencies. Furthermore, although different in amplitude, the signals from both sensors have similar trends, indicating the feasibility of the low-cost sensor for detecting PD, as shown in the next section.

### 5.2. Analysis of PD Signals

**Figure 6:**
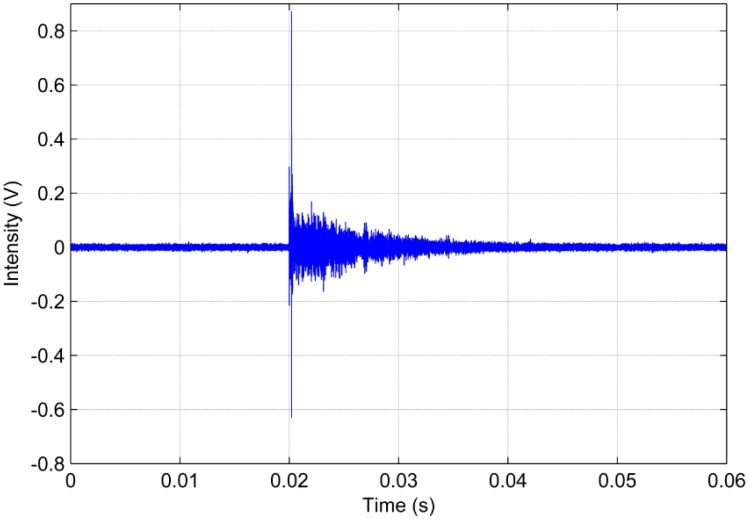
Partial discharge signal in the time domain obtained for the piezoelectric diaphragm.

**Figure 7:**
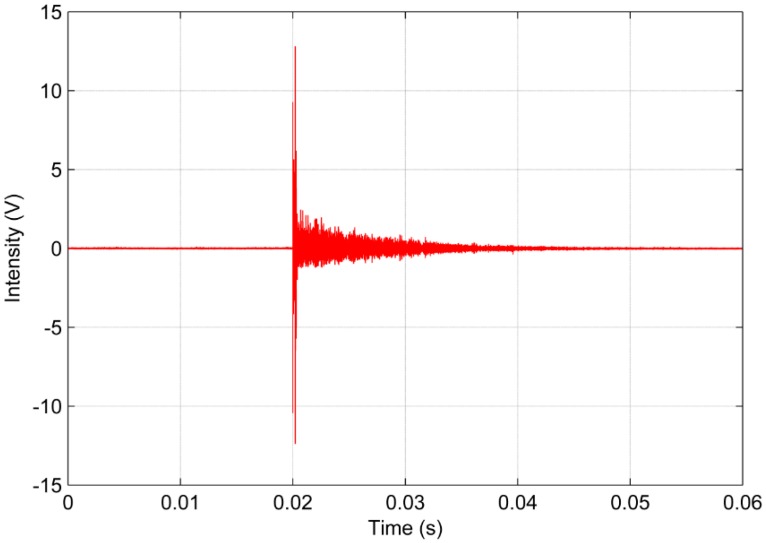
Partial discharge signal in the time domain obtained for the conventional AE sensor.

The analysis of PD signals was performed in the time and frequency domain using some criteria such as energy and RMS criteria, as previously defined, beyond the correlation analysis. Figure 6 and Figure 7 show the signals in the time domain of the piezoelectric diaphragm and the conventional AE sensor, respectively.

Observing Figure 6 and Figure 7, there is a significant difference in amplitude between the two waveforms in the time domain. In general, the conventional AE sensor provides a signal with an amplitude approximately 15 times higher than the low-cost sensor. However, despite the discrepancy in amplitude, both signals have very similar trends. Both signals show abrupt variations at approximately the time of 0.02 s, clearly indicating the occurrence of the PD phenomenon, and then exhibit a similar decreasing trend. Therefore, these results indicate the feasibility of low-cost piezoelectric diaphragms for detecting PDs under the experimental conditions considered in this study.

**Figure 8:**
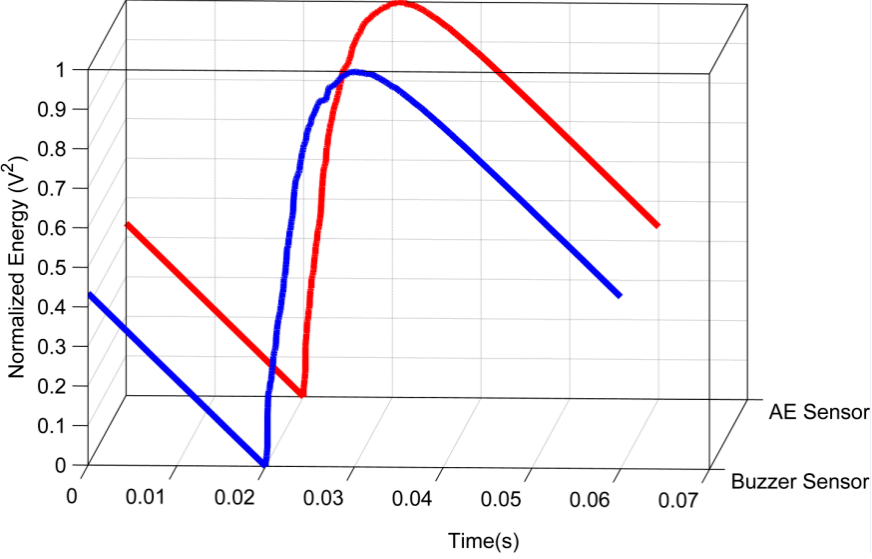
Energy signals of the sensors for partial discharge.

A more appropriate comparison of the behavior of the signals from the two sensors can be performed by computing the energies, as shown in Figure 8.

The energy signals were normalized between the values 0 and 1 to allow a satisfactory comparison because the signals have different amplitudes, as shown in the previous results. According to Figure 8, the energy signals are very similar, indicating a high correlation between them. The energy signal from the both sensors increases at the instant approximately of 0.02 s when the PD occurs and then progressively decreases. The results are consistent with the signals shown in Figure 6 and Figure 7.

**Figure 9:**
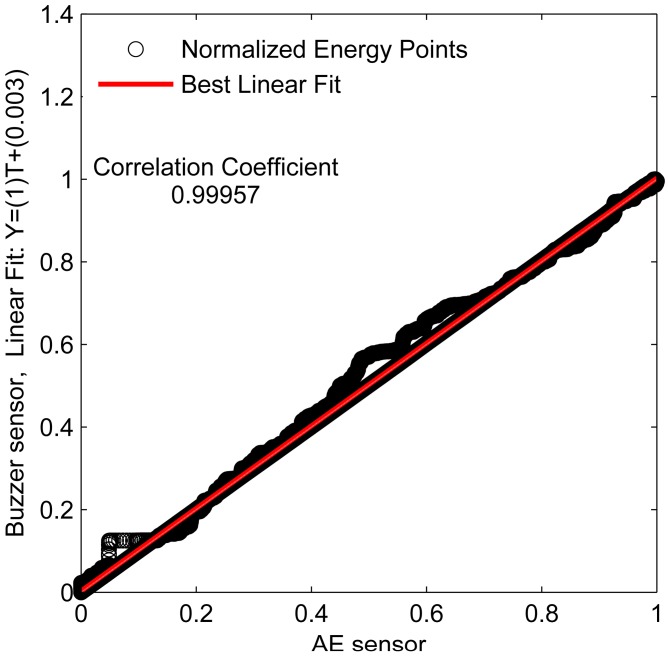
Linear fit and correlation coefficient for the energy.

A linear approximation was performed and the correlation coefficient was calculated to show the similarity of the behavior of the signals obtained for the both sensors, as shown in Figure 9.

**Figure 10:**
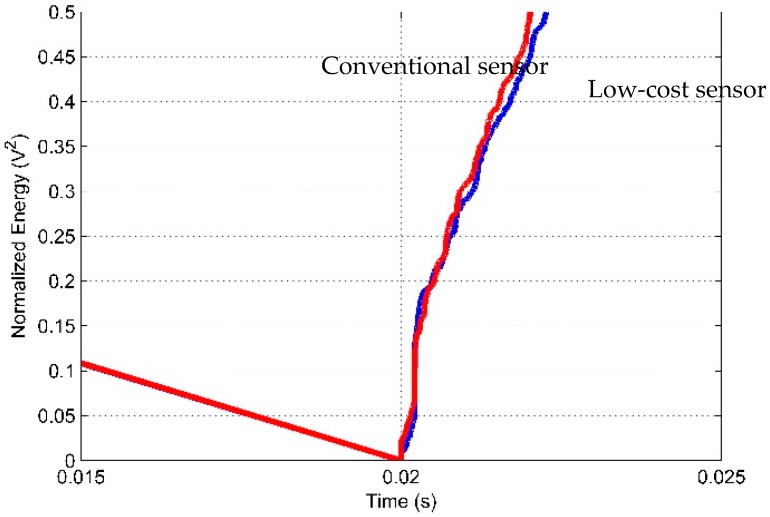
Time of arrival of acoustic waves.

The correlation coefficient of the energy signals is equal to 0.99957, i.e., the linear fit is close to 45°, which indicates a high similarity between both energy signals. The energy criterion provides the TOA of the acoustic waves, as shown in Figure 10. Figure 10 shows that the TOA is the same for both sensors (*t* = 0.02 s). Therefore, the there are no delay between the signals from the piezoelectric diaphragm and the conventional AE sensor. 

**Figure 11:**
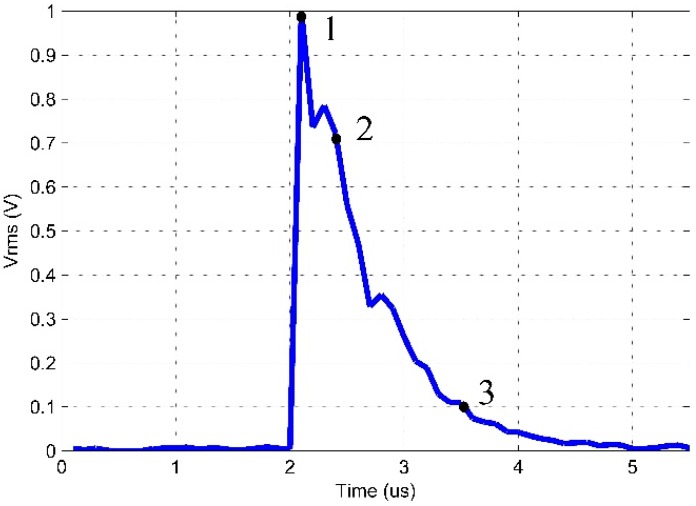
RMS values of the partial discharge signal obtained for the piezoelectric diaphragm.

**Figure 12:**
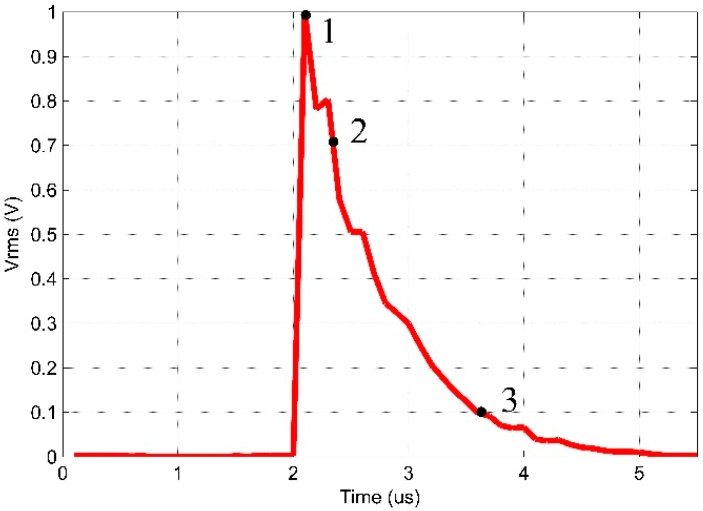
RMS values of the partial discharge signal obtained for the conventional AE sensor.

Figure 11 and Figure 12 show the RMS values of the AE signals obtained for the low-cost piezoelectric sensor and the conventional AE sensor. The window period value considered for such criterion is 1 ms. This approach is related to the intensity of vibration and the load applied.

The three points highlighted in Figure 11 and Figure 12 and labeled as 1, 2 and 3 represent the RMS peak value, an attenuation point with 70.7% of the RMS peak value (3 dB attenuation) and an attenuation point with 10% of the RMS peak value, respectively. These points were chosen for the comparison of the signals. Table 2 shows the features of these points. 

The results indicate the similarity between the two signals. At the time of 2 µs, the RMS voltage varied significantly from 0 to 1 V (peak) during 0.1 µs for both sensors. Table 2 shows that the peak value obtained by the sensors occurred at the same time (*t* = 2.1 µs). The attenuation of 3 dB (70.7% of the RMS peak value) occurred at 2.4 µs and 2.3 µs for the piezoelectric diaphragm and the conventional AE sensor, respectively. For the 10% of the RMS peak value, the attenuation occurred at 3.6 µs for the diaphragm and 3.5 µs for the conventional AE sensor, i.e., a difference of 0.1 µs. Therefore, the behaviors of both sensors are similar regarding to the PD applied. 

**Figure 13:**
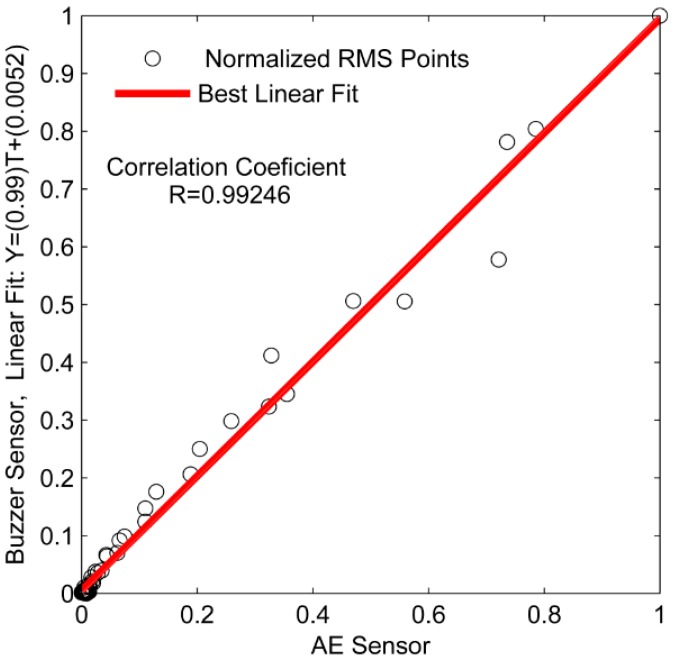
Linear fit and correlation coefficient for RMS signals.

Figure 13 shows this similarity where the angle coefficient of curve is close to 45°, indicating a high correlation between the signals obtained for the both sensors, since that the correlation coefficient is 0.999246, i.e., very close to 1.

**Figure 14:**
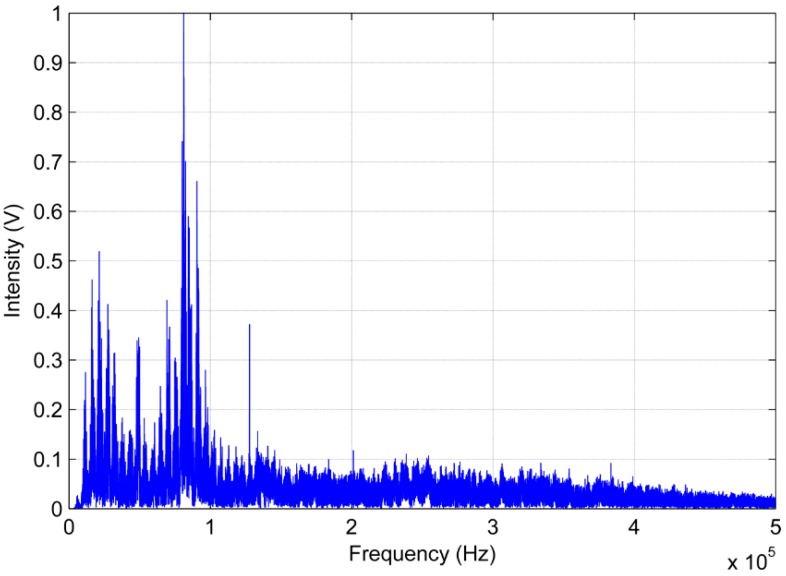
Frequency response of the piezoelectric diaphragm.

**Figure 15:**
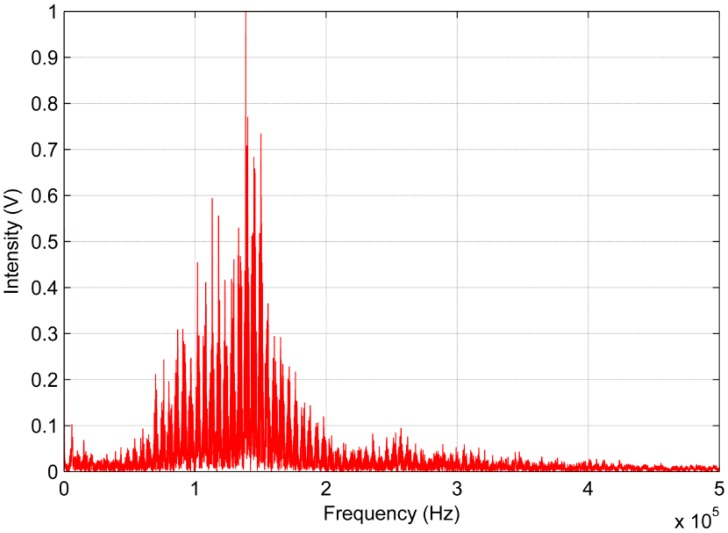
Frequency response of the conventional AE sensor.

The frequency response analysis of the AE signals was performed using the FFT algorithm. Figure 14 and Figure 15 show the frequency response analysis.

In regarding to the analysis of the AE signals generated by the PD in the frequency domain, there is a discrepancy with respect to regions of greater sensitivity shown by both sensors. Figure 14 shows that the low-cost sensor gets a significant sensitivity to frequencies below 120 kHz. Moreover, it can also be observed that the frequency of the peak wave provided by FFT is approximately 90 kHz for the low-cost sensor. In contrast, the conventional AE sensor obtained a better response frequency between 90 kHz to 250 kHz. For the conventional sensor, the frequency of peak wave is approximately 140 kHz, as shown in Figure 15. 

**Figure 16:**
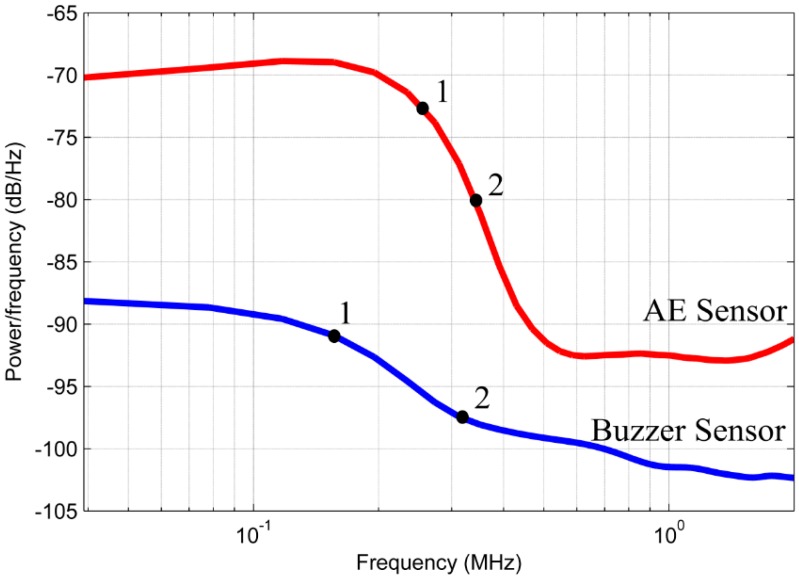
Power spectral density obtained for the low-cost and the conventional sensor.

Figure 16 shows the PSDs obtained for the two sensors, in which two points were highlighted to perform a quantitative comparison. Table 3 shows the frequencies and attenuations of the PSDs at each highlighted point. 

The analysis of power in the frequency domain shows that the low-cost sensor has an attenuation of 3 dB at 160 kHz and an attenuation of 10 dB is observed at 310 kHz. In contrast, the conventional AE sensor presented an attenuation of 3 dB at 260 kHz and an attenuation of 10 dB at 340 kHz. Although the bandwidth for the piezoelectric diaphragm is smaller than the conventional sensor, the bandwidth was similar when both sensors have 10 dB attenuation considering a difference of 30 kHz. Figure 14, Figure 15 and Figure 16 show that the PD spectrum is more significant up to 250 kHz and it reaches very low intensity peaks to frequencies above 250 kHz. 

Finally, it is important to note that the relative lower sensitivity of the low-cost sensor compared with the conventional AE sensor observed in Figure 5 and Figure 16 can be improved by using an appropriate amplifier. The use of an appropriate amplifier must be taken into account for actual field applications.

## 6. Conclusions

The purpose of this study was to analyze the feasibility of piezoelectric diaphragms (buzzers) for detecting partial discharges in power transformers based on the acoustic emission method. The piezoelectric diaphragms have a low cost compared to the conventional sensors and are readily available, making the use of these devices particularly attractive in applications where several sensors are employed.

Several tests were carried out in order to compare the two sensors. The experimental results indicate that the low-cost sensor has lower sensitivity compared to the conventional sensor. However, the signals obtained for the two sensors have similar behaviors, indicating the feasibility of the low-cost sensor for detecting partial discharges under the experimental conditions considered in this study.

It is important to note that the results presented in this study are only valid under the experimental conditions evaluated in the tests. The partial discharge simulated in the tests is relatively strong compared to the transformer tank size. In addition, the tests were carried out under optimal laboratory conditions. Therefore, future research considering different intensities of partial discharges and larger transformers, the influence of temperature, external disturbances and noise, it is still required for a more complete assessment of the use of low-cost piezoelectric diaphragms for detecting partial discharges in field applications, such as in power transformers in substations.

## Figures and Tables

**Table 1:** Attenuation and frequency of the points highlighted in Figure 5.

Sensor	Point	Attenuation (dB)	PSD (dB/Hz)	Frequency (kHz)
**Diaphragm**	1	3	104.6	120
	2	10	111.6	215
**Conventional**	1	3	98.5	237
	2	10	105.5	357

**Table 2:** Points highlighted in Figure 11 and Figure 12.

Point	RMS Peak (Vrms)	Diaphragm Sensor Time (µs)	Conventional Sensor Time (µs)
**1**	100%	2.1	2.1
**2**	70.7%	2.4	2.3
**3**	10%	3.6	3.5

**Table 3:** Frequency and attenuation of each point highlighted in Figure 16.

Sensor	Point	Attenuation (dB)	PSD (dB/Hz)	Frequency (kHz)
**Diaphragm**	1	3	91.5	160
	2	10	98.15	310
**Conventional**	1	3	73.21	260
	2	10	80.21	340
